# Raman Spectroscopy Enables Non-Invasive Identification of Peanut Genotypes and Value-Added Traits

**DOI:** 10.1038/s41598-020-64730-w

**Published:** 2020-05-07

**Authors:** Charles Farber, Lee Sanchez, Stanislav Rizevsky, Alexei Ermolenkov, Bill McCutchen, John Cason, Charles Simpson, Mark Burow, Dmitry Kurouski

**Affiliations:** 1 Department of Biochemistry and Biophysics, Texas A&M University, College Station, Texas 77843 United States; 2 Department of Biotechnology, Binh Duong University, Thu Dau Mot, 820000 Vietnam; 3 Texas A&M AgriLife Research, Stephenville, Texas 76401 United States; 4 Texas A&M AgriLife Research, Lubbock, Texas 79403 United States; 5 The Institute for Quantum Science and Engineering, Texas A&M University, College Station, Texas 77843 United States

**Keywords:** Imaging and sensing, Applied optics, Plant breeding

## Abstract

Identification of specific genotypes can be accomplished by visual recognition of their distinct phenotypical appearance, as well as DNA analysis. Visual identification (ID) of species is subjective and usually requires substantial taxonomic expertise. Genotyping and sequencing are destructive, time- and labor-consuming. In this study, we investigate the potential use of Raman spectroscopy (RS) as a label-free, non-invasive and non-destructive analytical technique for the fast and accurate identification of peanut genotypes. We show that chemometric analysis of peanut leaflet spectra provides accurate identification of different varieties. This same analysis can be used for prediction of nematode resistance and oleic-linoleic oil (O/L) ratio. Raman-based analysis of seeds provides accurate genotype identification in 95% of samples. Additionally, we present data on the identification of carbohydrates, proteins, fiber and other nutrients obtained from spectroscopic signatures of peanut seeds. These results demonstrate that RS allows for fast, accurate and non-invasive screening and selection of plants which can be used for precision breeding.

## Introduction

Continuous growth of the global population requires perpetual increase in the production of food. It is expected that by 2050 we will need to produce 70% more food^1^. Such expectations can be met only though major transformations in currently used agricultural approaches. For instance, utilization of sensor-based field irrigation in 50% of ornamental operations can save up to 223 billion liters of water per year in the U.S. alone, or the water use of approximately 400,000 U.S. households^2^. Satellite or unmanned aerial vehicle (UAV) guided imaging can be used to monitor agricultural crops to minimize harvest losses. This new agricultural paradigm, also known as digital farming, aims to automate agricultural processes via application of precision location (GPS) methods and artificial intelligence.

Although many processes in modern agriculture have reached a substantial degree of automation, plant breeding and taxonomic identification are far from that point. Currently, to positively identify a plant, visual inspection or genotyping are the only options. The first approach is subjective and typically requires substantial knowledge and practical experience. Years of training may be required to train a plant breeder or a botanist to be the expert in one area of the plant kingdom. To exclude the human factor in plant identification, Baena *et al*. used RGB imaging from UAVs to identify plants^3^. However, the reported results demonstrated that such approach may work only for large plants with major morphological differences. Genotyping, whether by sequencing or other methods, is broadly employed by scientists and breeders to identify the genetic material associated with traits of interest. However, these methods are destructive, time-consuming and labor-intensive.

Raman spectroscopy (RS) is a label-free, non-invasive and non-destructive analytical technique that can be used to probe chemical composition of analyzed samples. Our group recently demonstrated that RS can be utilized for confirmatory diagnostics of biotic and abiotic stresses on plants. Specifically, we showed that using a hand-held Raman spectrometer, one can diagnose several devastating fungal diseases on corn, wheat and sorghum with high accuracy^4,5^. We also demonstrated that RS can be used to pre-symptomatic diagnostics of citrus greening disease of orange and grapefruit trees and pests inside cowpea seeds^6,7^. Additionally, we showed that RS could be used to diagnose rose rosette diseases on roses^8^. These studies demonstrated that RS can detect chemical changes in plants and potentially revolutionize agriculture.

RS could potentially enable simultaneous digital phenotyping and nutrient assessment of plant materials. This would pave the way for autonomous seed selection, or automatic breeding. Additionally, should RS be able to detect genotypic, disease-associated, and nutrient-related changes in plants, it could be deployed to provide real-time health information for plants in greenhouses or out in the field.

In this proof of principle study, we demonstrate the potential of RS for the accurate identification of peanut genotypes based on chemometric analysis of their leaves and seeds. Additionally, we demonstrate that RS can be used to screen peanut leaves for nematode resistance as well as oleic-to-linoleic acid content ratios. Finally, we show that RS can probe the relative contents of carbohydrates, fiber oils, proteins, fatty acids and esters in these same peanut seeds.

## Results and Discussion

### Leaf-based spectrotyping of peanuts

**Figure 1:**
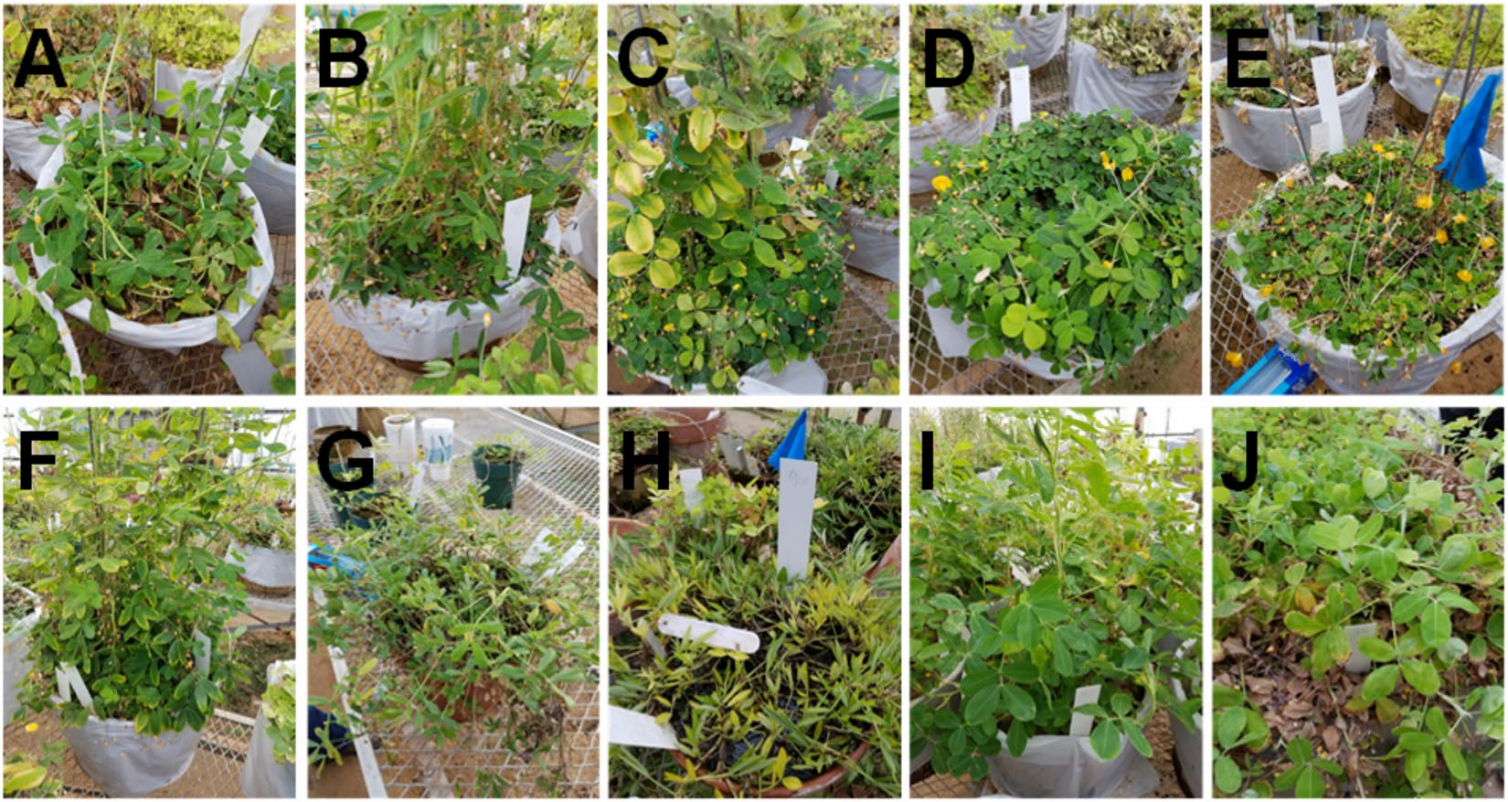
Phenotypical appearance of ten different peanut genotypes (**A–J**) used for the study, identities are as follows: (**A**) *Arachis archeri* (PI475987), (**B**) *Arachis sp*. (36009), (**C**) *Arachis cardenasii* (36035Y), (**D**) *Arachis helodes* (6331-3), (**E**) *Arachis kulmannii* (7631-1), (**F**) *Arachis matiensis* (36007), (**G**) *Arachis nitida* (S-3942), (**H**) *Arachis subcoriacae* (13706-1), (**I**) *Arachis hypogaea* (TP 623-1-2), (**J**) *Arachis pintoi* (12787).

**Figure 2:**
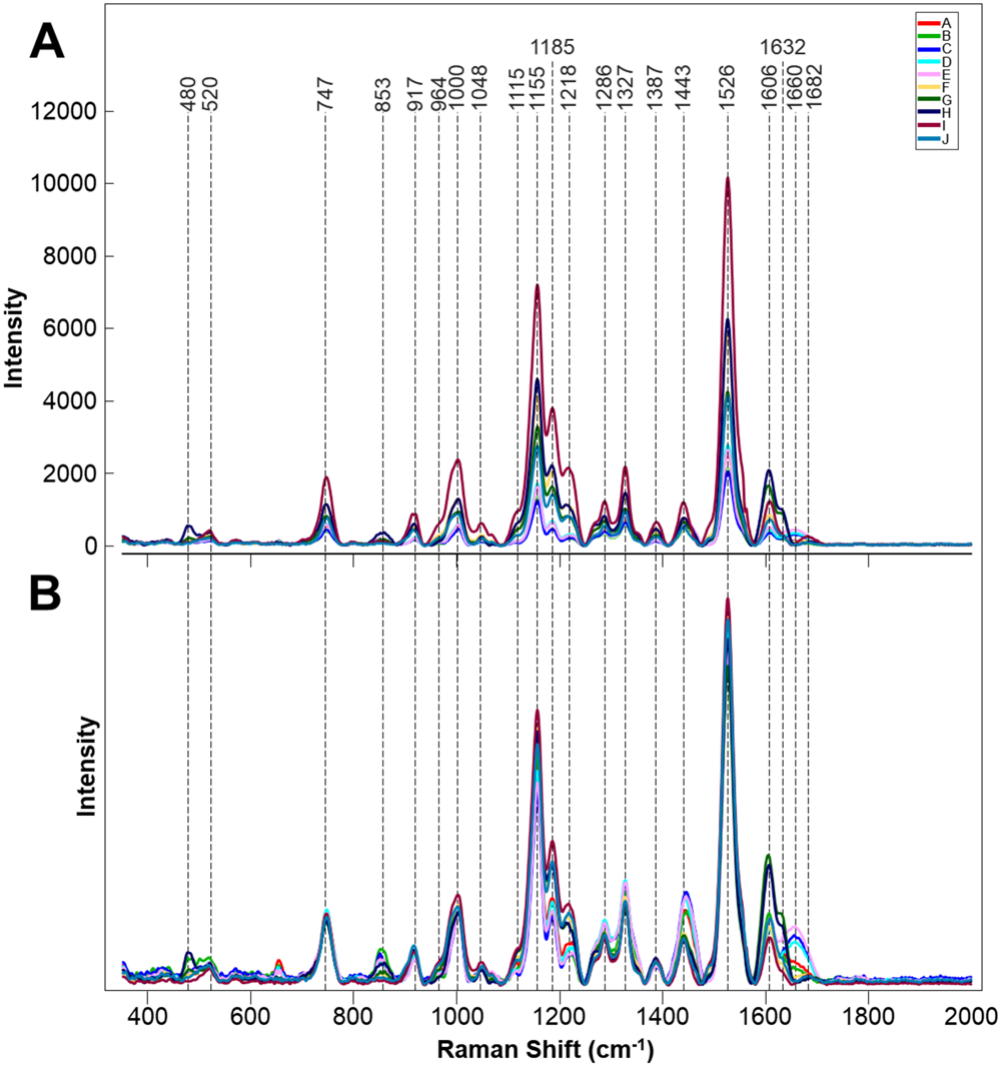
Averages of non-normalized (**A**) and area normalized (**B**) Raman spectra collected from leaves of 10 different peanut genotypes.

Raman spectra collected from the 10 different genotypes of peanuts (Fig. 1) exhibited similar profiles with vibrational bands at 480, 917 cm^−1^, which can be assigned to carbohydrates, 520 and 1048 and 1115 cm^−1^ to cellulose, 747 and 853 cm^−1^ to pectin, 1000, 1155 and 1526 cm^−1^ to carotenoids, 1185, 1606 and 1632 cm^−1^ to phenylpropanoids (including lignin), 1660 cm^−1^ to proteins and 1682 cm^−1^ to carboxylic acids. We also observed vibrational bands at 964, 1286, 1327, 1387, 1443, which can be assigned to aliphatic groups (CH_2_/CH_3_ vibrations) (Fig. 2, Table 1).

**Table 1:** Vibrational bands and their assignments for spectra collected from leaves and seeds of peanuts.

Band (cm^−1^)	Vibrational mode	Assignment
480	C-C-O and C-C-C Deformations; Related to glycosidic ring skeletal deformations δ(C-C-C) + τ(C-O) Scissoring of C-C-C and out-of-plane bending of C-O	Carbohydrates^22^
520	ν(C-O-C) Glycosidic	Cellulose^23^
747	γ(C–O-H) of COOH	Pectin^24^
849–853	(C6–C5–O5–C1–O1)	Pectin^25^
917	ν(C-O-C) In plane, symmetric	Cellulose, phenylpropanoids^23^
964–969	δ(CH_2_)	Aliphatics^26,27^
1000–1005	In-plane CH_3_ rocking of polyene aromatic ring of phenylalanine	Carotenoids^28^; protein
1048	ν(C-O) + ν(C-C) + δ(C-O-H)	Cellulose, phenylpropanoids^23^
1080	ν(C-O) + ν(C-C) + δ(C-O-H)	Carbohydrates^22^
1115–1119	Sym ν(C-O-C), C-O-H bending	Cellulose^23^
1155	C-C Stretching; v(C-O-C), v(C-C) in glycosidic linkages, asymmetric ring breathing	Carotenoids^28^,carbohydrates^29^
1185	ν(C-O-H) Next to aromatic ring + σ(CH)	Phenylpropanoids^30,31^
1218	δ(C-C-H)	Aliphatics^26^, xylan^30^
1265	Guaiacyl ring breathing, C-O stretching (aromatic); -C=C−	Phenylpropanoids^32^, unsaturated fatty acids^19^
1286	δ(C-C-H)	Aliphatics^26^
1301	δ(C-C-H) + δ(O-C-H) + δ(C-O-H)	Carbohydrates^22,33^
1327	δCH_2_ Bending	Aliphatics, cellulose, phenylpropanoids^23^
1339	ν(C-O); δ(C-O-H)	Carbohydrates^22^
1387	δCH_2_ Bending	Aliphatics^26^
1443–1446	δ(CH_2_) + δ(CH_3_)	Aliphatics^26^
1526	-C=C- (in plane)	Carotenoids^34,35^
1606–1632	ν(C-C) Aromatic ring + σ(CH)	Phenylpropanoids^36,37^
1654–1660	-C=C-, C=O Stretching, amide I	Unsaturated fatty acids^19^, proteins^34^
1682	COOH	Carboxylic acids
1748	C=O Stretching	Esters, aldehydes, carboxylic acids and ketones^38^

Identification of nematode resistance and Raman-based selection for high O/L ratios.

We employed orthogonalized partial least squares discriminant analysis (OPLS-DA) to determine whether RS can be used for the quantitative identification of peanut genotypes based on spectroscopic signatures collected from their leaflets. The loading plot and misclassification table were then generated using this final model, which contained 9 predictive components (PCs), one orthogonal component and 1021 (690–1710 cm^−1^) original wavenumbers for standard normal variate (SNV) pre-processed first derivative spectra. The nine predictive components (PCs) explained a total of 43% variation between the classes (Fig. S1).

The model identified the carotenoids band at 1525 cm^−1^ (PC1), the phenylpropanoid bands at 1606 and 1632 cm^−1^ (PC1), cellulose bands at 1155 cm^−1^ (PC1), the xylan band at 1218 cm^−1^ (PC2), the hydrocarbons bond vibration at 1443 cm^−1^ (PC2), as well as the cellulose and phenylpropanoid band at 1185 cm^−1^ (PC3) as the strongest predictors of species. The model also explained 47% of the variation (R2X) in the spectra. Our results indicated that RS can be used for, on average, 80% accurate identification in of peanut plants based on their spectroscopic signatures from leaflets (Table 2). However, some varieties had markedly lower accuracy of classification than others. This may be associated with growth stage of the plants.

**Table 2:** OPLS-DA confusion matrix of Raman spectra collected from leaves of 10 different genotypes (A–J) of peanuts.

	Members	Correct	A	B	C	D	E	F	G	H	I	J
A	70	69%	48	1	4	2	0	1	0	1	13	0
B	70	83%	0	58	6	0	2	1	1	0	0	2
C	70	71%	3	1	50	0	10	2	0	2	0	2
D	70	63%	1	1	4	44	5	7	0	1	6	1
E	69	70%	1	6	1	0	48	1	1	0	5	6
F	70	66%	2	2	1	1	2	46	1	3	12	0
G	70	91%	0	0	0	0	0	0	64	2	1	3
H	70	99%	0	0	0	0	0	0	1	69	0	0
I	71	100%	0	0	0	0	0	0	0	0	71	0
J	72	88%	0	0	1	0	0	0	2	0	6	63
Total	702	80%										

**Figure 3:**
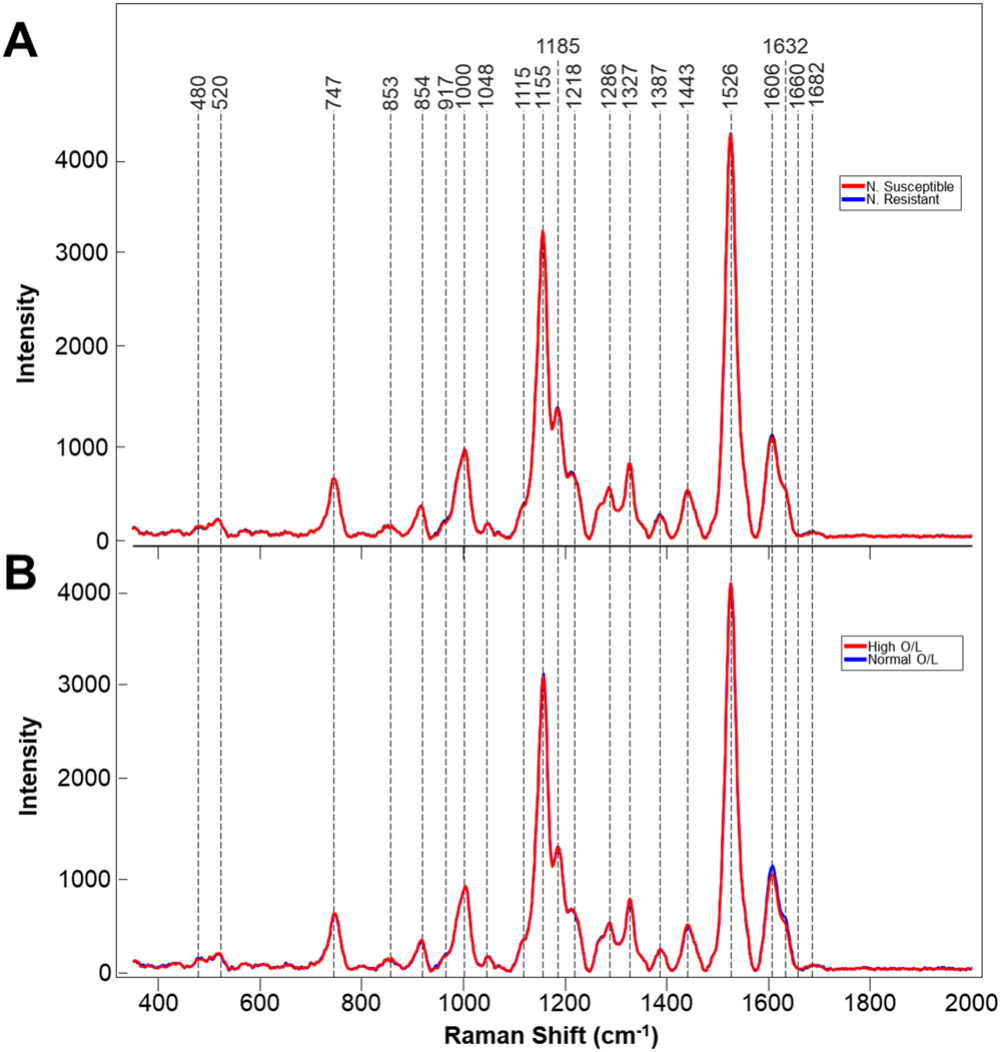
Averaged Raman spectra collected from the leaves of: (**A**) nematode(N) resistant and susceptible plants or (**B**) plants with differing O/L ratios in their seeds.

Resistance was previously identified in wild species peanut that imparted almost total immunity to root-knot nematode (*Meloidogyne arenaria*)^9^. Several highly resistant cultivars have been released from the resulting gene introgression^10–12^. Root-knot nematode resistance is associated with a failure of juveniles to establish a feeding site^13^. We looked at whether RS can be used to probe for resistance in peanut cultivars. Although the average spectroscopic signatures of nematode resistant and susceptible plants are very similar (Fig. 3A), partial least squares discriminant analysis (PLS-DA) allowed for on average 75% accurate identification of these two groups, Table 3. From the loadings plot (Fig. S2), we observed that changes associated with the carotenoid (1526 cm^−1^), phenylpropanoid (1606 cm^−1^) and the 1155 cm^−1^ bands as the most important for this prediction. Further biochemical analysis is required to determine the relationship between these compounds in the leaves and nematode resistance may be.

**Table 3:** PLS-DA cross-validation confusion matrix of Raman spectra collected from leaves of nematode resistant and susceptible peanut varieties.

	Members	Correct	Nematode resistant	Nematode susceptible
Nematode resistant	197	71.1%	140	51
Nematode susceptible	241	78.8%	57	190
Total	438	74.9%		

Next, we employed RS to distinguish between plants that were known to have a high and normal oleic (O/L) ratio. High-oleic peanuts are preferred by manufactures because they have a longer shelf life which leads to reduced rancidity, and have been shown to be associated with reduced serum cholesterol levels and reductions in cardiovascular disease^14^. The following high-oleic varieties have been officially released by Texas A&M AgriLife as plant variety protected cultivars: Tamrun OL01^12^, Tamrun OL02^15^, Tamrun OL06^15^, Tamrun OL07^15^, Webb^11^, Tamrun OL11^16^, Schubert^17^, and TamVal OL14^18^. We collected more than 300 spectra from peanut varieties and breeding lines with either high (23 breeding lines and 1 released cultivars) and low O/L ratios (4 released cultivars). RS revealed that high O/L plants have lower phenylpropanoid content (1606 and 1632 cm^−1^), whereas structure of all other structural components of these two groups of plants appeared to be nearly identical. (Fig. 3B). PLS-DA of these spectra allowed for identification of high vs normal O/L plants with an average 82% accuracy, Table 4. Inspecting the loadings plot (Fig. S3), we found that the important bands for O/L prediction were quite like those for nematode prediction, as the plot patterns are very similar. However, in O/L, it can be observed that the phenylpropanoid bands are associated with the first latent variable (LV) in O/L, whereas they are observed in the third LV in nematode resistance. As each LV explains successively less variation in a dataset, this may suggest that phenylpropanoid content is more important for prediction of O/L content than for nematode resistance. These results demonstrate that RS can be used to assist plant breeders by allowing for fast screening of genetic traits.

**Table 4:** PLS-DA cross-validation confusion matrix of Raman spectra collected from leaves of peanut varieties with high and low O/L ratios.

	Members	Correct	Low O/L	High O/L
Low O/L	77	77.9%	60	33
High O/L	241	86.3%	17	208
Total:	318	82.1%		

### Seed-based identification of peanuts and assessment of their nutrient values

**Figure 4:**
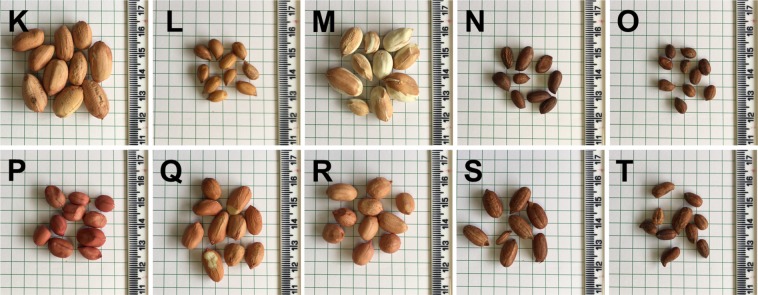
Photographs of seeds of ten different peanut genotypes (**K–O**). Identities are as follows: (**K**) *Arachis hypogaea* (TxL090106-05), (**L**) *Arachis valida* (30147), (**M**) *Arachis hypogaea* (US# 1551 Tan), (**N**) *Arachis praecox* (6416), (**O**) *Arachis cardenasii* (10017), (**P**) *Arachis hypogaea* (US# 1519 Red), (**Q**) *Arachis hypogaea* (TxAG-8), (**R**) *Arachis hypogaea* (Tx144932), (**S**) *Arachis gladulifera* (30098), (**T**) *Arachis paraguariensis* (10585).

**Figure 5:**
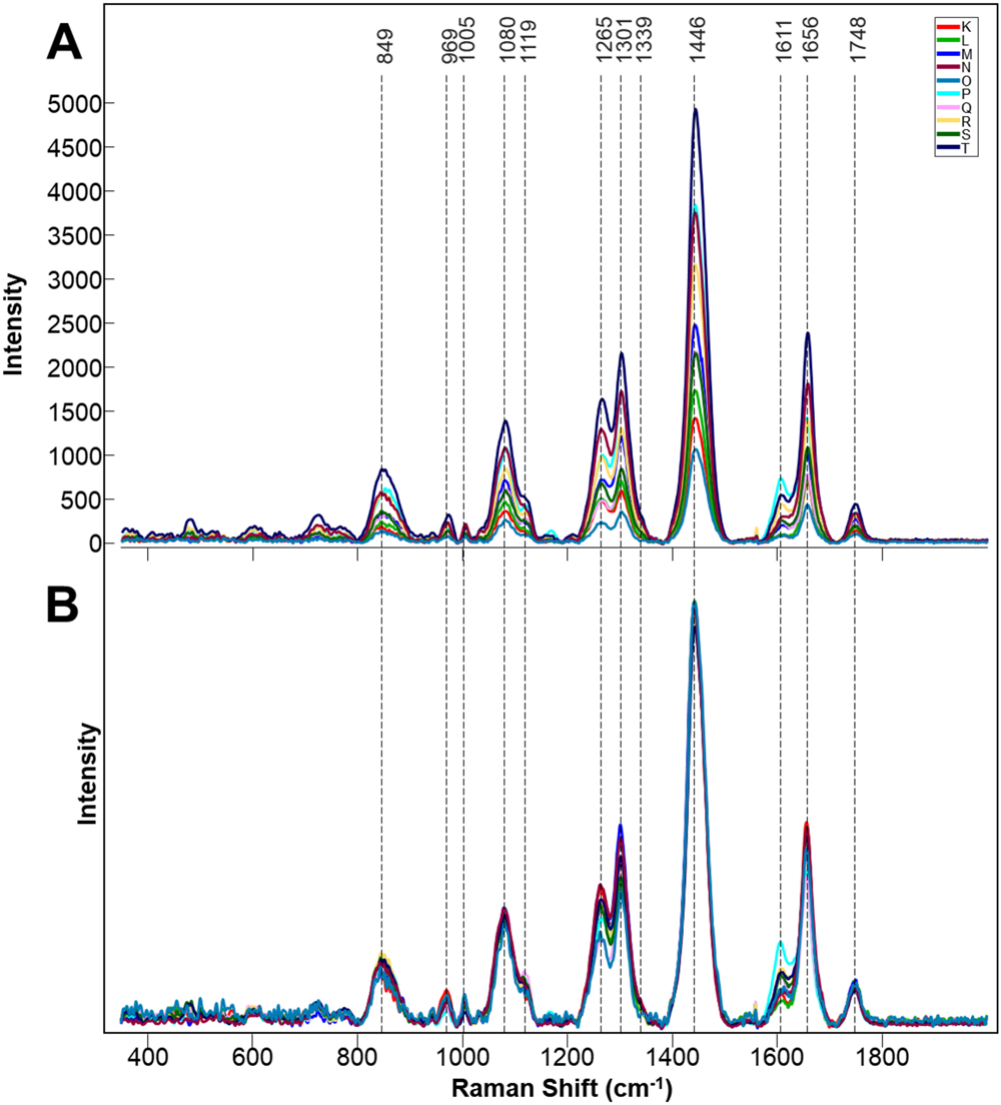
Averages of non-normalized (**A**) and area normalized (**B**) Raman spectra collected from seeds of 10 different peanut genotypes.

We also investigated whether RS can be used to positively identify peanut seeds. We collected 342 spectra from 5 wild (assigned K through O) and 5 cultivated (assigned P through T) genotypes of peanuts (Fig. 4). The Raman spectra of peanut seeds exhibited vibrational bands that could be assigned to pectin (849 cm^−1^), carbohydrates (1080, 1119, 1301, 1339 cm^−1^), and phenylpropanoids (1611 cm^−1^) (Fig. 5). We also observed vibrational bands at 969 and 1446 cm^−1^, which can be assigned to CH_2_ and CH_2_/CH_3_ vibrations respectively. The vibrational band at 1005 cm^−1^ could potentially be assigned to both carotenoids and proteins. In addition to the vibrational band at ~1000 cm^−1^, carotenoids also have a distinct vibrational band around 1530 cm^−1^ which was not observed in the Raman spectra of peanut seeds. Therefore, we can unambiguously assign the vibrational band at 1005 cm^−1^ to proteins. The vibrational band at 1656 cm^−1^ can be also assigned to proteins (amide I). However, this band may also originate from -C=C- vibrations of unsaturated fatty acids. The -C=C- group also exhibits the vibrational mode at 1265 cm^−1^, which was observed in the Raman spectra of peanut seeds^19^. Since intensities of 1265 and 1656 cm^−1^ change synchronously from one genotype to another, it is highly likely that both of them should be assigned to the same chemical moiety. Therefore, the vibrational band at 1265 cm^−1^ has been assigned to the alkene group of unsaturated fatty acids. Finally, the vibrational band at 1748 cm^−1^ can be assigned to esters.

Next, we investigated whether these Raman spectra of peanut seeds can be used to probe nutrient composition. The intensity of vibrational bands in Raman spectra directly correlate with a concentration of the corresponding chemical in the sample. Therefore, we can use intensities of 1005, 1301, 1443, and 1607 cm^−1^ vibrational bands to probe relative content of proteins, carbohydrates, oils and fiber, as well as vibrational bands at 1656 and 1748 cm^−1^ to predict the amount of unsaturated fatty acids and esters in peanut seeds. Previously, we showed that overall spectral intensity directly depends on the color of the sample. For instance, in our study on the nutrient content of corn kernels, spectra collected from dark colored corn kernels had much lower intensity comparing to the Raman spectra collected from lighter color kernels^20^. Therefore, to exclude the influence of seed color on the intensities of vibrational bands, spectra were normalized to the total spectral area, as this method is the least biased.

**Figure 6:**
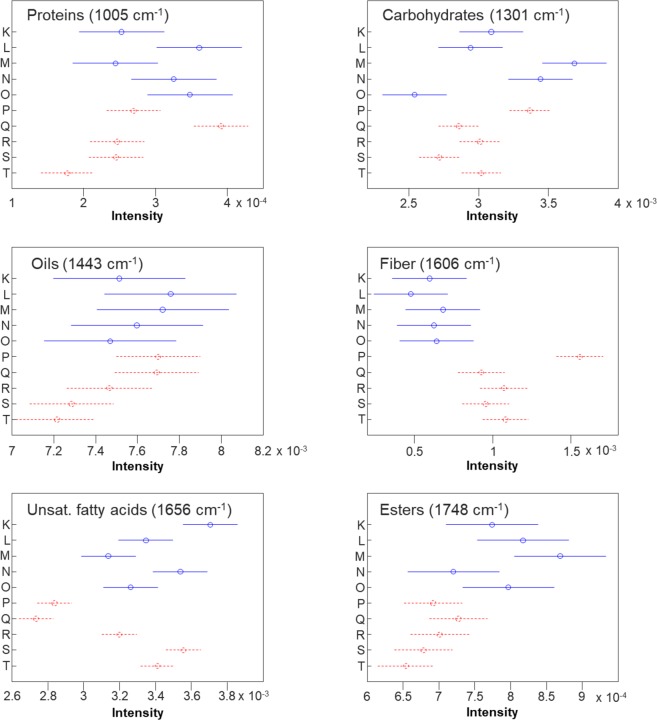
Means (circles) and 95% confidence intervals for the intensities of the peanut seed spectra, normalized to total spectral area, at the indicated wavenumbers. Generated following the ANOVA tests. Blue and solid: wild variety; Red and dashed: cultivated variety.

We used ANOVA to determine whether the previously analyzed differences in nutrient-associated bands were statistically significant (Fig. 6). In general, we found that the wild varieties tended to have wider confidence intervals for the true mean intensity of a given band of interest compared to the cultivated varieties. This is logical as the cultivated varieties have been bred for specific traits of interest. At the protein band (1005 cm^−1^; Fig. 6, top left), ANOVA revealed that varieties L, O and Q have significantly higher protein band intensity than R, S and T. Additionally, variety N has significantly higher intensity than variety T. Finally, variety Q is significantly more intense than all varieties except L, N and O. Variety Q may have been selectively bred for higher protein content compared to the other varieties, while within the wild varieties, none of the groups significantly differ from each other.

The carbohydrate band varied as well (Fig. 6, top right). Varieties M, N and P are significantly more intense than all other cultivated varieties. The remaining cultivated varieties (Q – T) do not have significantly different intensities from each other. Variety O is significantly less intense than varieties K, M, N and P. The general trends observed for protein remain true for carbohydrates: the cultivated varieties have more narrow confidence intervals, which is probably due to being selected for traits associated with carbohydrate buildup.

In terms of oil content (Fig. 6, middle left), varieties L, M, P and Q have significantly higher intensity than variety T but are not different from each other. In fact, Additionally, varieties P and Q have significantly higher intensity than S and T, which suggests that these varieties may be bred for higher oil content. All other varieties are not significantly different from each other. In fiber content (Fig. 6, middle right), the centers of the wild variety confidence intervals were all lower than the centers of the cultivated varieties. However, only varieties P, R and T have significantly higher fiber-associated intensity than the wild cultivars. Variety P is significantly more intense than all other varieties analyzed, a grouping which only appears once in all bands analyzed.

Additionally, we discovered that for unsaturated fatty acids (Fig. 6, bottom left), varieties P and Q are significantly less intense than all other varieties. Varieties K, N, S and T are significantly more intense than variety R, and variety K is significantly more intense than all other varieties but N and S. Finally, in esters (Fig. 6, bottom right), we found that while only M and N are significantly different in terms of within category (wild and cultivated) differences, variety M is significantly more intense than all cultivated varieties. In fact, the centers of the confidence intervals for the cultivated varieties are all lower than those of the wild varieties. Nevertheless, only K, L, M, and O are significantly more intense than the least intense cultivated variety T.

We then used OPLS-DA to demonstrate that RS can be used for identification of peanuts genotypes based on the spectra collected from their seeds. The loading plot (Fig. S4) and misclassification table (Table 5) were then generated using this final model, which contained 9 predictive components, one orthogonal component and 1100 (701–1800 cm^−1^) original wavenumbers for SNV pre-processed first derivative spectra. The nine predictive components (PCs) explained a total of 55% variation between the classes.

**Table 5:** OPLS-DA confusion matrix of Raman spectra collected from seeds of 10 different genotypes (K-T) of peanut.

	Members	Correct	K	L	M	N	O	P	Q	R	S	T
K	15	93%	14	0	0	0	0	0	1	0	0	0
L	20	95%	0	19	0	0	0	0	0	1	0	0
M	14	93%	0	0	13	0	1	0	0	0	0	0
N	20	100%	0	0	0	20	0	0	0	0	0	0
O	50	94%	0	0	0	0	47	0	3	0	0	0
P	49	96%	0	0	0	0	0	47	0	0	2	0
Q	50	100%	0	0	0	0	0	0	50	0	0	0
R	50	90%	0	0	0	0	0	0	0	45	5	0
S	54	96%	0	0	0	0	0	1	0	1	52	0
T	20	95%	0	0	0	0	0	1	0	0	0	19
Total	342	95%										

The model identified the ester band at 1748 cm^−1^ (PC1), a phenylpropanoid band at 1611 cm^−1^ (PC1), a pectin band at 849 cm^−1^ (PC1), carbohydrate bands at 1302 and 1339 cm^−1^ (PC2), the hydrocarbons bond vibration at 1446 cm^−1^ (PC2), as well as unsaturated fatty acid bands at 1265 and 1656 cm^−1^ (PC3) as the strongest predictors of genotype. The model also explained 33% of the variation (R2X) in the spectra.

Our results demonstrated that RS can be used for highly accurate (95%) identification of peanut seeds. This accuracy is much higher than the positive identification of leaves. This can be explained by the very low if any metabolism occurring in the seeds as compared to actively growing plants.

## Conclusions

Our results demonstrate that RS can be used for accurate identification of peanuts based on spectroscopic signatures of their leaves and seeds. We showed that accuracy of plant identification is higher upon spectroscopic analysis of seeds comparing to leaves. OPLS-DA results show that peanut seeds can be identified with on average 95% accuracy whereas accuracy of Raman-based identification of leaves is, on average, 80%. These results demonstrate that RS can be used in field and greenhouses settings for rapid phenotyping of plants. We also showed that utilization of RS allows for non-invasive and non-destructive assessment of nutrient content of seeds providing information about their carbohydrate, protein, fiber, as well as oils and unsaturated fatty acids for peanut seeds. As this method is fast (1 s), portable, non-invasive and non-destructive, the reported experimental evidence suggests that RS can be used directly on combines and elevators for on-line monitoring of seed quality.

## Methods

### Leaves

Approximately one hundred leaflets were collected from 10 different genotypes of peanut plants grown in the greenhouse at the Texas A&M AgriLife Research and Extension Center at Stephenville 125 days after planting (DAP). Greenhouses are controlled by a Wadsworth Step-50 temperature control system in an IBG greenhouse. The system operated where the heaters cycle on if the temperature drops below 21 °C and the cooling system cycles on if the temperature exceeds 32 °C. To minimize the contribution from possible differences in plant vegetation stages, leaves were collected twice with approximately five weeks gap between sample collection. Spectra from both sampling rounds were used together in the statistical analysis. Up to four spectra were acquired per leaflet, based on their size.

Peanut leaflets were collected at 109 DAP on 10/14/19 from field plots at the Texas A&M AgriLife Research and Extension Center in Stephenville. Susceptible materials included 3 released cultivars and 23 breeding lines from the Texas A&M AgriLife peanut breeding program that do not contain the gene introgression from wild peanut. Resistant material included 1 released cultivar and 6 breeding lines from the program. We collected more than 700 Raman spectra from leaves of both nematode resistant and susceptible peanut varieties and breeding lines.

### Seeds

10 different genotypes of peanut seeds were provided from the Texas A&M AgriLife Research peanut germplasm collection. Seeds were removed from −20 °C short term cold storage and allowed to equilibrate to room temperature for approximately 24 hrs before scanning. 10–35 seeds per genotype were scanned two times each for use in variety differentiation by RS.

### Raman spectroscopy

#### Acquisition

Spectra were collected using a portable, hand-held Agilent Resolve spectrometer equipped with an 830 nm laser. The following experimental parameters were used for all collected spectra: 1 s acquisition time, 495 mW power, and surface scanning mode. Leaves and seeds were each gently pressed against the nose cone during scanning to ensure that they are at the focus of the laser.

#### Processing

Spectra were automatically background subtracted and baseline corrected by the instrument’s onboard software. These data were then exported from the portable instrument as comma separated value (CSV) files using software provided by the company. Raw spectra of leaves and seeds can be found in the SI as Figs S5 and S6, respectively.

These csv’s were then imported into MATLAB and SIMCA for preprocessing. Spectra were first normalized to unit variance using the standard normal variate (SNV) method in order to reduce the contribution of random changes in total spectral intensity. Then, all spectra were mean centered, which involves subtracting the mean spectrum from each individual spectrum. This process allows our analyses to be relative to the total mean of the sample. Statistical analyses were then conducted in SIMCA14, MATLAB, or PLS_Toolbox, an addon of MATLAB.

### Statistical analysis

#### PLS-DA

Spectra were imported into either SIMCA 14 (Umetrics, Umea, Sweden) or MATLAB for multivariate statistical analysis. To build classification models, partial least squares discriminant analysis (PLS-DA) was selected. PLS-DA is an extension of ordinary PLS which uses dummy Y-variables to indicate discrete classes or categories of data which the model then proceeds to predict^21^. PLS-DA is a type of supervised learning model, meaning that the user must provide the categories for each datapoint during training. Once the model completes training, it is then cross validated: part of the dataset is excluded while the rest is used to train the model. The model then attempts to predict the class membership of the excluded datapoints. This process is repeated until every datapoint has been excluded. In this study, we chose to report cross-validation results, which are suggestive of the model’s ability to classify unseen data. Each PLS-DA model will contain predictive components (PCs), also known as latent variables (LVs), each of which explain a percentage of the variation in the dataset. In orthogonal PLS-DA, variation in the data is separated into a predictive portion which accounts for separation between classes, and an orthogonal portion which does not.

Differentiation of peanut varieties using leaf or seed spectra was conducted in SIMCA 14 using OPLS-DA and preprocessing described in the main text. Differentiation of nematode and O/L content was conducted in the MATLAB addon PLS_Toolbox (Eigenvector Research Inc.) using regular PLS-DA.

#### Anova

We used analysis of variance (ANOVA) to screen our peanut samples for their nutrient content. ANOVA is a statistical procedure which tests whether any means in a set of samples are significantly different from each other. The null hypothesis of this test is that there are no significant differences amongst the categories being tested. A significant (α = 0.05) ANOVA indicates that at least one pair of groups have significantly different means. To determine which groups were significantly different from each other, we then conducted Tukey HSD tests that evaluate which groups are significantly different. We then report 95% confidence intervals for the true value of each mean. Overlapping confidence intervals indicate that those groups are not significantly different from each other.

To conduct ANOVAs on our peanut nutrient dataset, spectra were first imported into MATLAB. They were then normalized to total spectral area and intensities at individual wavenumbers (Fig. 6) were extracted. ANOVA was then conducted using the anova1 function on the intensities at each of the selected wavenumbers. 95% confidence intervals were constructed using the multcompare function, which by default uses Tukey HSD to evaluate group-to-group differences.

## Supplementary information

Supplementary information.

## References

[1] Food and Agriculture Organization of the United Nations. How to feed the world 2050. (2009).

[2] Majsztrik JC, Price EW, King DM (2013). Environmental benefits of wireless sensor-based irrigation networks: Case-study projections and potential adoption rates. HortTechnology.

[3] Baena S, Moat J, Whaley O, Boyd DS (2017). Identifying species from the air: Uavs and the very high resolution challenge for plant conservation. PLoS One.

[4] Egging V, Nguyen J, Kurouski D (2018). Detection and identification of fungal infections in intact wheat and sorghum hrain using a hand-held raman spectrometer. Anal. Chem..

[5] Farber, C. & Kurouski, D. Detection and identification of plant pathogens on maize kernels with a hand-held raman spectrometer. *Anal. Chem*., 10.1021/acs.analchem.8b00222 (2018). 10.1021/acs.analchem.8b00222 29461798

[6] Sanchez L, Farber C, Lei J, Zhu-Salzman K, Kurouski D (2019). Noninvasive and nondestructive detection of cowpea bruchid within cowpea seeds with a hand-held raman spectrometer. Anal Chem.

[7] Sanchez, L., Pant, S., Xing, Z., Mandadi, K. & Kurouski, D. Rapid and noninvasive diagnostics of huanglongbing and nutrient deficits on citrus trees with a handheld raman spectrometer. *Anal Bioanal Chem*, 10.1007/s00216-00019-01776-00214 (2019). 10.1007/s00216-019-01776-4 30989272

[8] Farber C, Shires M, Ong K, Byrne D, Kurouski D (2019). Raman spectroscopy as an early detection tool for rose rosette infection. Planta.

[9] Nelson SC, Simpson CE, Startt JL (1990). Expression of resistance to meloidogyne arenaria in arachis batizicoi and a. Cardenasii. j. Nematol..

[10] Simpson CE, Starr JL (2001). Registration of ‘coan’ peanut. Crop. Sci..

[11] Simpson CE (2013). Registration of ‘webb’ peanut. J. Plant Resist.

[12] Simpson CE, Starr JL, Church GT, Burow MD, Paterson AH (2003). Registration of ‘nematam’ peanut. Crop. Sci..

[13] Timper P, Holbrook CC, Xue HQ (2000). Expression of nematode resistance in plant introduction of arachis hypogaea. Peanut Sci.

[14] Allen AH (2008). Priority areas for research on the intake, composition, and health effects of tree nuts and peanuts. J. Nutr.

[15] Baring MR (2007). Registration of ‘tamnut ol06′ peanut. Crop. Sci..

[16] Baring MR, Simpson CE, Burow MD, Cason JM, Ayers JL (2013). Registration of ‘tamrun ol11′ peanut. J. Plant Resist.

[17] Burow MD (2016). Registration of ‘schubert’ peanut. J. Plant Resist.

[18] Burow MD (2019). Registration of “tamval ol14 peanut. J. Plant Resist.

[19] Jamieson LE, Li A, Faulds K, Graham D (2018). Ratiometric analysis using raman spectroscopy as a powerful predictor of structural properties of fatty acids. R Soc Open Sci.

[20] Krimmer M, Farber C, Kurouski D (2019). Rapid and noninvasive typing and assessment of nutrient content of maize kernels using a handheld raman spectrometer. ACS Omega.

[21] Eriksson, L., Byrne, T., Johansson, E., Trygg, J. & Vikstrom, C. *Multi- and megavariate data analysis basic principles and applications*. 3rd revised edn, Vol. 1 500 (Umetrics, 2013).

[22] Almeida MR (2010). Determination of amylose content in starch using raman spectroscopy and multivariate calibration analysis. Anal. Bioanal. Chem..

[23] Edwards HG, Farwell DW, Webster D (1997). Ft raman microscopy of untreated natural plant fibres. Spectrochim. Acta A.

[24] Synytsya A, Čopíková J, Matějka P, Machovič V (2003). Fourier transform raman and infrared spectroscopy of pectins. Carbohydr. Polym.

[25] Engelsen SB, Nørgaard L (1996). Comparative vibrational spectroscopy for determination of quality parameters in amidated pectins as evaluated by chemometrics. Carbohydrate Polymers.

[26] Yu MM, Schulze HG, Jetter R, Blades MW, Turner RF (2007). Raman microspectroscopic analysis of triterpenoids found in plant cuticles. Appl. Spectrosc..

[27] Cabrales L, Abidi N, Manciu F (2014). Characterization of developing cotton fibers by confocal raman microscopy. Fibers.

[28] Schulz H, Baranska M, Baranski R (2005). Potential of nir-ft-raman spectroscopy in natural carotenoid analysis. Biopolymers.

[29] Wiercigroch E (2017). Raman and infrared spectroscopy of carbohydrates: A review. Spectrochim. Acta A.

[30] Agarwal UP (2014). 1064 nm ft-raman spectroscopy for investigations of plant cell walls and other biomass materials. Front. Plant. Sci..

[31] Mary YS, Panicker CY, Varghese HT (2012). Vibrational spectroscopic investigations of 4-nitropyrocatechol. Orient. J. Chem.

[32] Cao Y, Shen D, Lu Y, Huang J (2006). A raman-scattering study on the net orientation of biomacromolecules in the outer epidermal walls of mature wheat stems (triticum aestivum). Ann. Bot.

[33] Cael JJ, Koenig JL, Blackwell J (1975). Infrared and raman spectroscopy of carbohydrates. 4. Normal coordinate analysis of v-amylose. Biopolymers.

[34] Devitt G, Howard K, Mudher A, Mahajan S (2018). Raman spectroscopy: An emerging tool in neurodegenerative disease research and diagnosis. ACS Chem. Neurosci.

[35] Adar F (2017). Carotenoids - their resonance raman spectra and how they can be helpful in characterizing a number of biological systems. Spectroscopy.

[36] Kang L, Wang K, Li X, Zou B (2016). High pressure structural investigation of benzoic acid: Raman spectroscopy and x-ray diffraction. J. Phys. Chem. C..

[37] Agarwal UP (2006). Raman imaging to investigate ultrastructure and composition of plant cell walls: Distribution of lignin and cellulose in black spruce wood (picea mariana). Planta.

[38] Colthup, N. B., Daly, L. H. & Wiberley, S. E. *Introduction to infrared and raman spectroscopy*. 3rd edn, (Academic Press, 1990).

