# Supplementary material for: Raman Spectroscopy Enables Non-Invasive Identification of Peanut Genotypes and Value-Added Traits
Source: Sci Rep. 2020 May 7;10:7730. doi: 10.1038/s41598-020-64730-w (PMC7206150; doi:10.1038/s41598-020-64730-w)
Supplement: Supplementary file 1 — Supplementary information. [file 41598_2020_64730_MOESM1_ESM.docx]

Raman Spectroscopy Enables Non-Invasive Identification of Peanut Genotypes and Value-Added Traits

Charles Farber^1^, Lee Sanchez^1^, Stanislav Rizevsky^1,2^, Alexei Ermolenkov^1^, Bill McCutchen^3,4^, John Cason^4^, Charles Simpson^4^, Mark Burow^5,6^ and Dmitry Kurouski^1,7^*

1. Department of Biochemistry and Biophysics, Texas A&M University, College Station, Texas 77843, United States
2. Department of Biotechnology, Binh Duong University Thu Dau Mot, 820000 Vietnam
3. Department of Entomology, Texas A&M University, College Station, Texas 77843, United States
4. Texas A&M AgriLife Research and Extension Center at Stephenville, Stephenville, Texas 76401, United States
5. Department of Soil and Crop Sciences, Texas A&M University, College Station, Texas 77843, United States
6. Texas A&M AgriLife Research and Extension Center at Lubbock, Lubbock, Texas 79403, United States
7. The Institute for Quantum Science and Engineering, Texas A&M University, College Station, Texas, 77843, United States

Corresponding Author: Dmitry Kurouski; E-mail: [dkurouski@tamu.edu](mailto:dkurouski@tamu.edu). Tel: 979-458-3778

**Supplemental Materials**

Figure S1. Loadings plots of the first three predictive components of the leaf genotype prediction model (Table 2).

Figure S2. Loadings plot for the first three latent variables of the nematode resistance model (Table 3).

Figure S3. Loadings plot for the first three latent variables of the O/L ratio model (Table 4).

Figure S4. Loadings plots of the first three predictive components of the leaf genotype prediction model (Table 5).

Figure S5. Raw, unprocessed (left) and processed (right) averaged leaf spectra obtained with the portable instrument.

Figure S6. Raw, unprocessed (left) and processed (right) averaged seed spectra obtained with the portable instrument.
